# A warm welcome to our new editors

**DOI:** 10.1002/2211-5463.12847

**Published:** 2020-04-07

**Authors:** Miguel A. De la Rosa

**Affiliations:** ^1^ FEBS Open Bio

## Abstract

In this Editorial, the Editor‐in‐Chief Professor Miguel A. De la Rosa introduces the new members of the Editorial Board and discusses the effects of COVID‐19 on the journal.

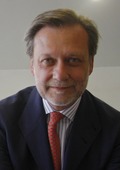

This month, I am delighted to announce the appointment of In Hye Lee, Josep Rizo, and Guang‐Biao Zhou to the editorial board of *FEBS Open Bio*. These appointments bring new expertise to the journal and also ensure that the journal is editorially represented in China, South Korea, and the United States. Biosketches of our new editors are included below. I am also pleased to announce the appointment of Janesh Kumar [National Centre for Cell Science (NCCS), Pune, India] and Chongde Sun [School of Agriculture and Biotechnology, Zhejiang University, China] to our Editorial Advisory Board. I welcome all of our new editors to the journal.

I would also like to take this opportunity to sincerely thank all of our editors and reviewers for their hard work and support at this very challenging time. The ongoing COVID‐19 crisis has caused massive disruption globally, creating challenges for us all. Increasing numbers of researchers are facing the disruption of laboratory closures and having to arrange online teaching while simultaneously dealing with the effects of the pandemic on their daily lives. Some of our editors, reviewers, and authors are also physicians battling the pandemic on the frontlines. As such, delays during this difficult period are unavoidable, and I would like to thank everyone for their kind understanding and patience. We are happy to give extensions to all authors who need it, even after deadlines have passed. Please contact the editorial office at openbio@febs.org if you require an extension for any reason.

## New members of the Editorial Board

### In Hye Lee

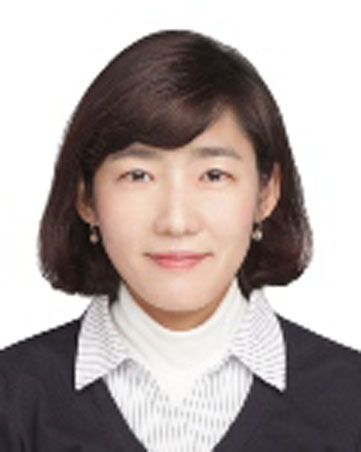

Dr. In Hye Lee completed her doctoral training as well as undergraduate studies at Ewha Womans University (EWU), Seoul, South Korea, where she studied cell signaling pathways to regulate cell proliferation, cell cycle, and tumorigenesis. After receiving her Ph.D. from EWU, she moved to the National Heart Lung Blood Institute (NHLBI), NIH, Bethesda, USA, for postdoctoral training in the laboratory of Dr. Toren Finkel, where she focused on the connection between autophagy and sirtuins. After being appointed as a federal government employer of the National Institute of Health (NIH), USA, she continued to study the physiological roles of autophagy. Since returning to EWU, Dr. Lee’s laboratory has focused on exploring molecular pathways to connect metabolism to aging.

### Josep Rizo

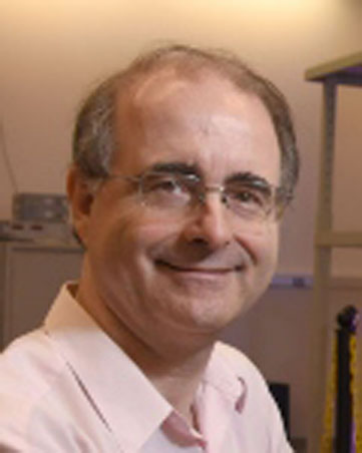

Professor Josep Rizo (Jose Rizo‐Rey) was born in Barcelona, Spain, in 1959. He received a B.Sc. degree in Organic Chemistry in 1981 from the University of Barcelona. During these college studies, he became very interested in quantum field theory and statistical mechanics, which led him to obtain a second B.Sc. degree, this time in theoretical physics, from the same university in 1988. During the 1982–1988 period, he also obtained MSc and PhD degrees in organic chemistry from the University of Barcelona, working in the laboratories of Ernest Giralt and Enrique Pedroso. He first worked on pioneering methodology to analyze peptides attached to solid supports by gel‐phase ^13^C NMR spectroscopy, and later on, the development of polar protecting groups for peptide synthesis. In 1989, he started postdoctoral research in the laboratory of Lila Gierasch at UT Southwestern, working on conformational studies of a variety of peptides by NMR spectroscopy and molecular dynamics. In the early 1990s, he shifted his interest to protein NMR and protein folding. He established his independent research group in 1995 at UT Southwestern. The main focus of his research is the study of the mechanisms of neurotransmitter release and intracellular membrane fusion using structural biology, a variety of biophysical techniques and reconstitution approaches. He is currently Professor in the Departments of Biophysics, Biochemistry, and Pharmacology.

### Guang‐Biao Zhou

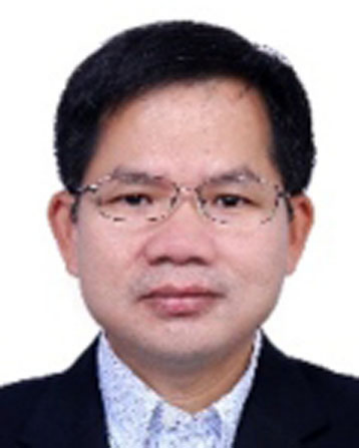

Dr. Guang‐Biao Zhou is Professor and Deputy Director of State Key Laboratory of Molecular Oncology, National Cancer Center/Chinese Academy of Medical Sciences Cancer Institute and Hospital. He was Distinguished Professor of the Chinese Academy of Sciences and is Director of the Department of Pathology and Pathophysiology of School of Medicine, University of Chinese Academy of Sciences. He investigates tobacco smoke‐ and air pollution‐induced lung carcinogenesis, including dissection of abnormalities in genomes, noncoding RNAs, immune escape, and inflammatory factors, using systematic approaches. He also investigates the mechanisms of action of traditional Chinese medicine in treating cancer. He has published more than 100 peer‐reviewed journal articles, and obtained the National Natural Science Funds for Distinguished Young Scholars from the National Natural Science Foundation of China. He is the Executive Deputy Editor‐in‐Chief of the journal *Frontiers of Medicine*. His suggestions to mitigate air pollution and protect people from air pollution‐induced health hazards were accepted by the government, resulting in an improvement of air quality in north China.

